# Extensive vertebral compression fractures as the initial manifestation of childhood B-cell acute lymphoblastic leukemia despite limited peripheral blood abnormalities: a case report

**DOI:** 10.3389/fped.2026.1945539

**Published:** 2026-10-12

**Authors:** Danmei Zou, Tong Zhou, Yilan Chen, Lianyu Wang, Xiaolan Xiao

**Affiliations:** 1 Guangzhou University of Chinese Medicine, Guangzhou, China; 2 Department of Pediatrics, The Second Affiliated Hospital of Guangzhou University of Chinese Medicine, Guangzhou, China

**Keywords:** acute lymphoblastic leukemia, bone marrow infiltration, case report, child, diagnostic delay, magnetic resonance imaging, vertebral compression fracture

## Abstract

**Background:**

Musculoskeletal manifestations may appear before hematologic abnormalities in childhood acute lymphoblastic leukemia (ALL), making diagnosis difficult. Extensive vertebral compression fractures involving the cervical, thoracic, and lumbar spine as the first sign of ALL are very rare, particularly when peripheral blood abnormalities are limited and circulating blasts are absent.

**Case presentation:**

We report a 5-year-old boy who presented with severe back pain and inability to walk after minor trauma. The initial lumbar computed tomography (CT) was reported as showing no definite vertebral fracture, but whole-spine magnetic resonance imaging (MRI) showed diffuse bone marrow infiltration with extensive pathological compression fractures involving the cervical, thoracic, and lumbar vertebrae. Peripheral blood tests showed leukopenia and neutropenia without circulating blasts, which contributed to the initial diagnostic uncertainty. Bone marrow examination confirmed common B-cell acute lymphoblastic leukemia with high hyperdiploidy and a KRAS p.G13D mutation. After induction therapy according to the South China Children's Cancer Group (SCCCG)-ALL-2023 protocol, his back pain was greatly relieved, he regained independent ambulation, and flow cytometric and molecular measurable residual disease (MRD) assessments became negative.

**Conclusion:**

Extensive vertebral compression fractures can be an early manifestation of childhood ALL, even when circulating blasts are absent and peripheral blood abnormalities are limited. Children with persistent axial skeletal pain or unexplained vertebral compression fractures should be evaluated for hematologic malignancy, especially when their symptoms are more severe than expected from routine imaging or peripheral blood findings. Early whole-spine MRI and timely bone marrow examination may help establish the diagnosis and avoid unnecessary delay.

## Introduction

Acute lymphoblastic leukemia (ALL) represents approximately one-quarter of childhood cancers and has diverse clinical presentations (1, 2). Musculoskeletal symptoms are common in childhood ALL and may appear before typical hematologic abnormalities (3, 4). Extensive vertebral compression fractures as the initial manifestation of ALL are rare (5). Such presentations may initially be attributed to orthopedic or rheumatologic diseases, delaying leukemia diagnosis and treatment (4). We report a child with common B-cell ALL who presented with extensive vertebral compression fractures despite limited peripheral blood abnormalities. The case highlights the importance of considering hematologic malignancy when severe skeletal symptoms are not adequately explained by the initial clinical and imaging findings.

## Case presentation

### Initial presentation

A 5-year-7-month-old boy with no previous medical problems developed severe lumbar pain and inability to walk after a minor fall on November 11, 2025. The next day, he developed sudden severe lumbar pain during defecation and could no longer stand or walk independently. The clinical course is summarized in Supplementary Table 1.

At a local hospital on November 12, 2025, lumbar CT showed no definite vertebral fracture or dislocation, and he was diagnosed with a soft-tissue injury. However, only the written radiological report from this examination was available, and the original CT images could not be retrieved for retrospective review. He received conservative treatment, but his back pain became worse. Two days later, he was taken to another hospital. Laboratory tests revealed leukopenia (white blood cell count 2.2 × 10⁹/L) with neutropenia (0.67 × 10⁹/L) and elevated high-sensitivity C-reactive protein (44.9 mg/L). Abdominal CT was performed at the referring hospital on November 15, 2025, with the lumbar spine included within the scanned field. Although the original CT examination was available for retrospective review, only axial images were accessible. A representative axial image at the L4 vertebral level is shown in Figure 1. Retrospective comparison at the corresponding vertebral level demonstrated that the vertebral abnormality was relatively inconspicuous on CT but more readily appreciable on MRI as abnormal marrow signal (Figure 1). Chest radiography, hip radiography, and brain MRI showed no obvious abnormalities.

**Figure 1:**
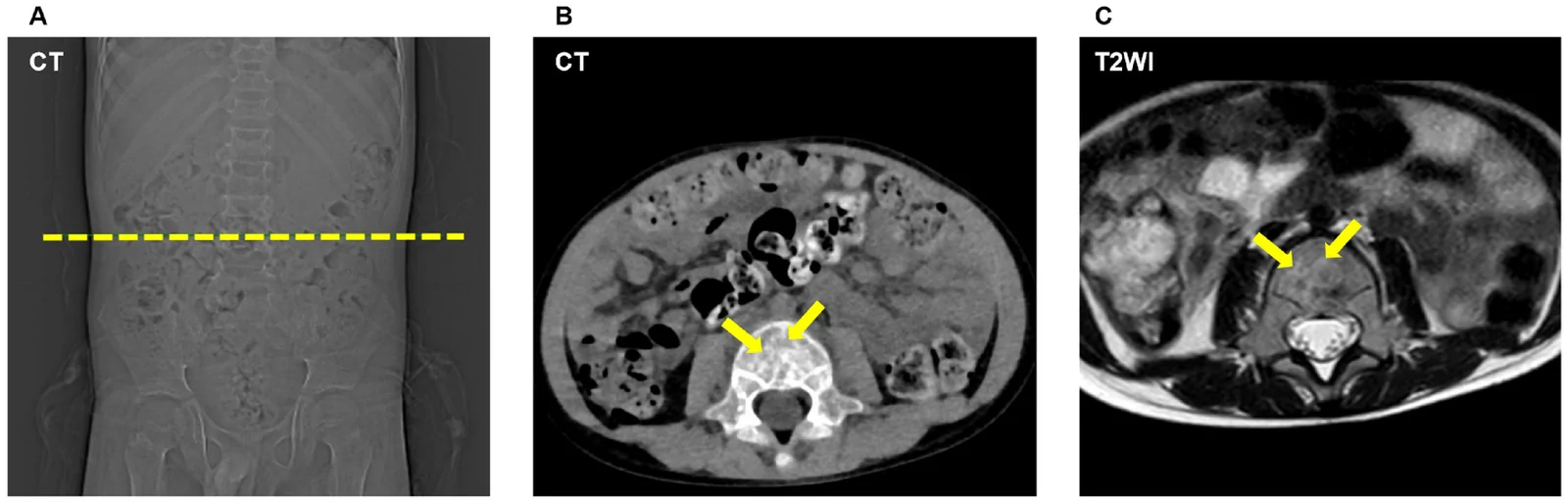
Retrospective comparison of CT and MRI findings at the L4 vertebral level. **(A)** CT scout image showing the lumbar spine; the yellow line indicates the level of the corresponding axial images. **(B)** Axial CT image at the L4 level, demonstrating heterogeneous vertebral attenuation with subtle irregular osseous changes (yellow arrow). **(C)** Corresponding axial T2-weighted MRI image at the L4 level, demonstrating heterogeneous abnormal vertebral marrow signal (yellow arrow).

The child received mezlocillin, oseltamivir, intravenous methylprednisolone (30 mg/day for 2 days), and supportive care. His back pain became less severe for a short time. Soon after, the pain worsened again, and he remained unable to walk. He was then admitted to our pediatric center for further evaluation on November 17, 2025.

About three months before admission, the child developed bilateral cervical and postauricular lymphadenopathy with a 2-day history of fever. Cervical ultrasonography showed multiple enlarged cervical and postauricular lymph nodes with preserved corticomedullary differentiation and preserved fatty hila. He received oral acyclovir, cefixime, and vitamin B complex. His symptoms improved after 3 days. He also had a history of glucose-6-phosphate dehydrogenase (G6PD) deficiency.

### Diagnostic evaluation

The patient had no fever on admission but was in obvious distress from severe back pain. Physical examination showed multiple enlarged cervical and postauricular lymph nodes on both sides, with the largest measuring up to approximately 3 cm. He had marked tenderness over the lumbosacral spine. Muscle strength in the lower limbs could not be assessed because of pain. Muscle tone, deep tendon reflexes, and the rest of the neurological examination were normal. He had no hepatosplenomegaly or sternal tenderness.

Initial blood tests showed persistent leukopenia and neutropenia (Figure 2), with no circulating blasts or dysplastic changes. Inflammatory markers remained mildly elevated. Antinuclear antibody (ANA) was positive at a titer of 1:320 with a speckled pattern, while Epstein–Barr virus serology indicated previous infection without evidence of active infection. Evaluation of mineral and bone metabolism showed a serum calcium level of 2.49 mmol/L, elevated phosphate of 2.31 mmol/L, alkaline phosphatase (ALP) of 141 U/L, and 25-hydroxyvitamin D [25(OH)D] of 45.5 nmol/L. Serum PTH and a dedicated acid-base assessment were not obtained.

**Figure 2:**
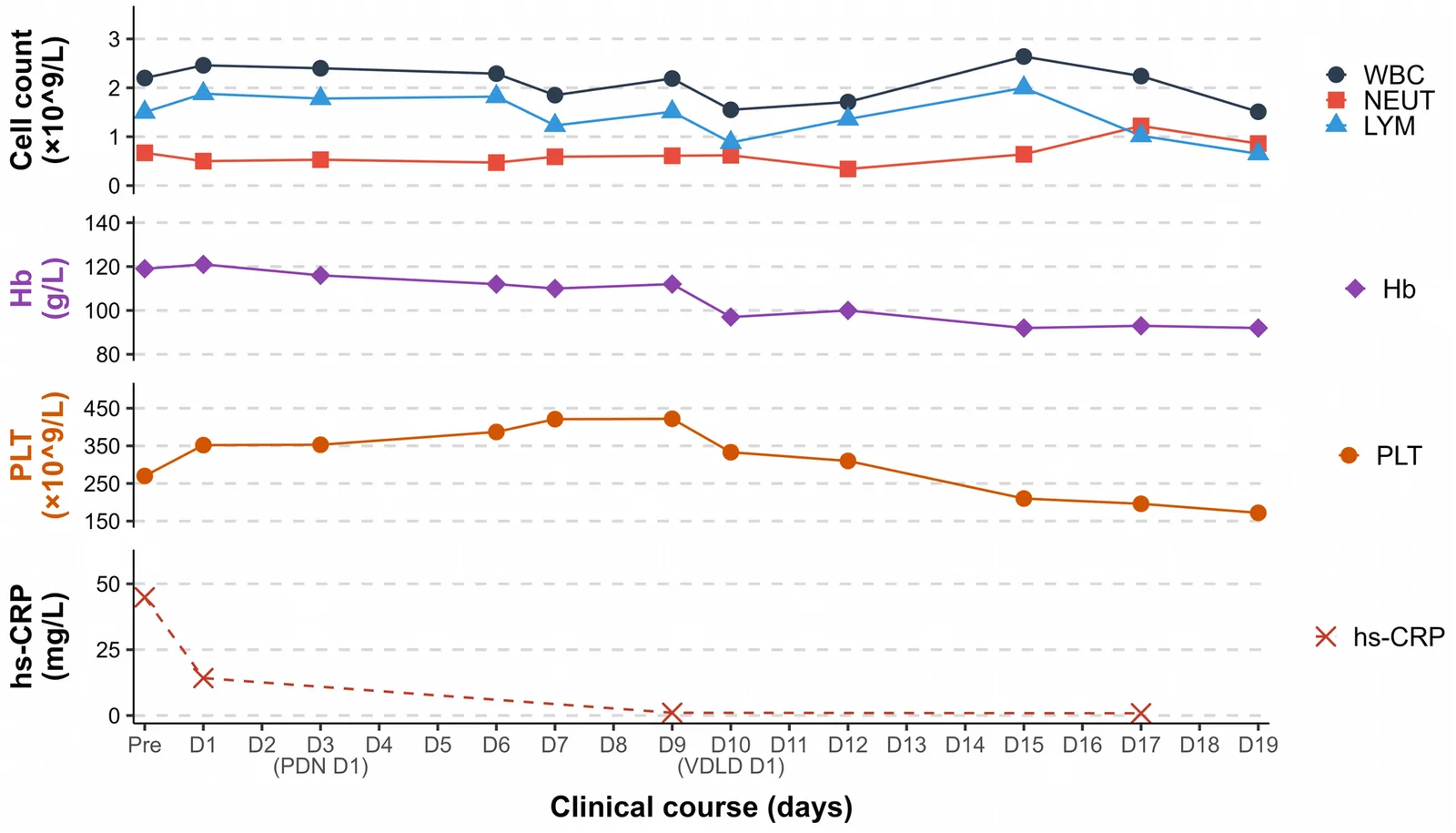
Dynamic changes in hematologic parameters and inflammatory markers during the early clinical course.

Serial laboratory parameters from initial presentation through the prednisone prephase (PDN) and induction chemotherapy (VDLD) are shown. The upper panel illustrates white blood cell count (WBC), absolute neutrophil count (NEUT), and lymphocyte count (LYM); the second panel shows hemoglobin concentration (Hb); the third panel shows platelet count (PLT); and the bottom panel shows high-sensitivity C-reactive protein (hs-CRP). PDN, prednisone prephase; VDLD, vincristine, daunorubicin, L-asparaginase, and dexamethasone (Figure 2).

His symptoms were much more severe than expected from the peripheral blood findings. Pediatric hematology, orthopedics, neurosurgery, and radiology specialists were consulted. Spinal infection remained a consideration because of the elevated inflammatory markers, but the vertebral abnormalities and persistent leukopenia/neutropenia prompted urgent bone marrow examination for suspected hematologic malignancy.

Bone marrow aspiration on hospital day 2 showed lymphoblasts in 59% of iliac and 71% of sternal marrow cells, supporting the diagnosis of ALL. Contrast-enhanced whole-spine MRI showed diffuse bone marrow infiltration and extensive pathological vertebral compression fractures involving the cervical, thoracic, and lumbar spine (Figure 3). Multiple vertebral bodies showed marked loss of vertebral body height, without spinal cord compression or epidural extension. Neck ultrasonography showed persistent cervical lymphadenopathy. Chest radiography showed mild inflammatory changes in the right upper lung. Other systemic imaging and bone mineral density testing were unremarkable.

**Figure 3:**
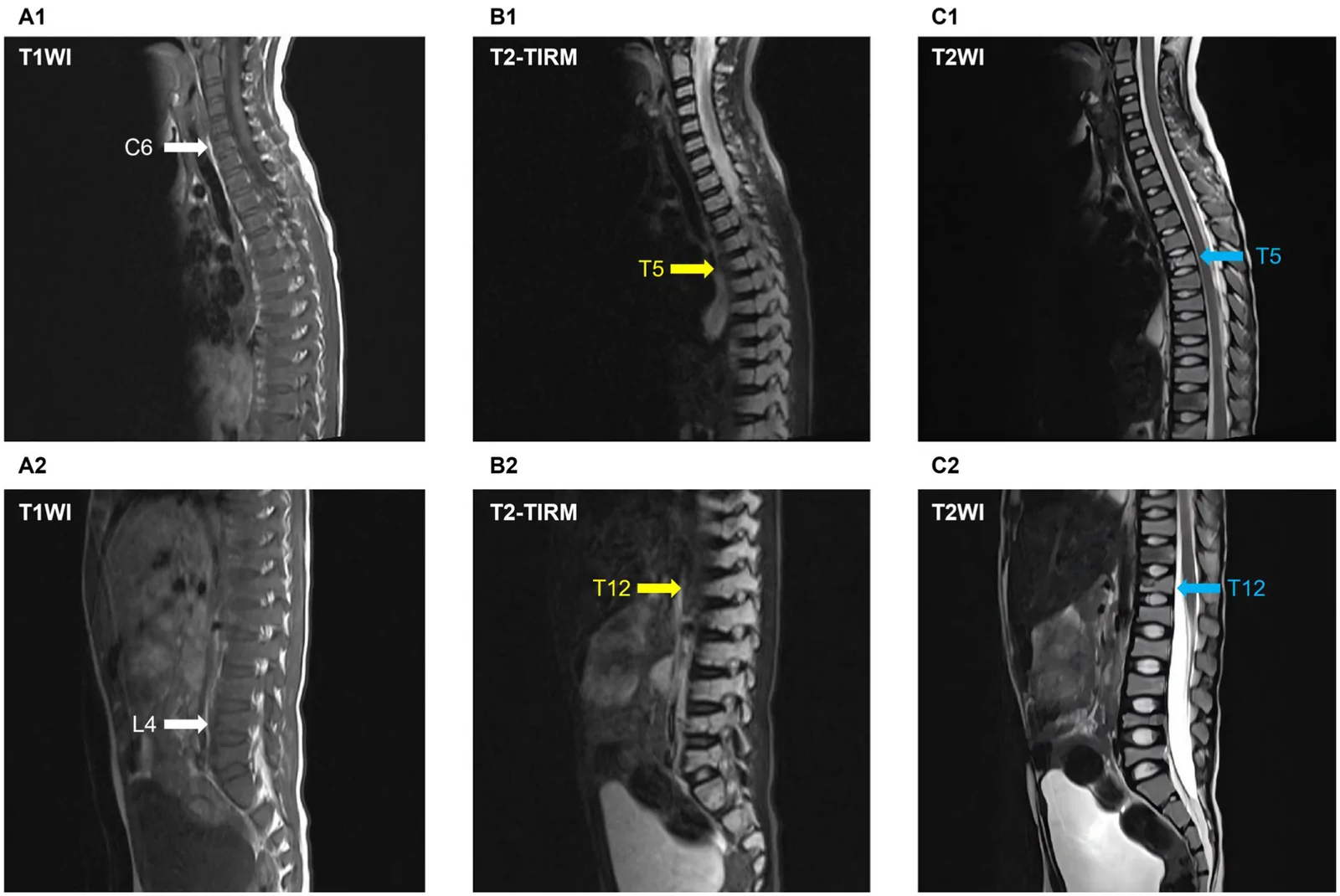
Whole-spine MRI demonstrating diffuse bone marrow infiltration and extensive pathological vertebral compression fractures at diagnosis.

Images are arranged according to spinal region (upper row, cervicothoracic spine; lower row, thoracolumbar spine) and MRI sequence (columns A–C). (A1–A2) Sagittal T1-weighted images show diffuse low marrow signal replacing normal fatty marrow (white arrows). (B1–B2) Sagittal T2-TIRM images show diffuse marrow hyperintensity with representative wedge compression fractures (yellow arrows). (C1–C2) Mid-sagittal T2-weighted images demonstrate preserved posterior vertebral cortex and spinal canal patency without spinal cord compression (blue arrows). Abbreviations: MRI, magnetic resonance imaging; T1WI, T1-weighted imaging; T2WI, T2-weighted imaging; TIRM, turbo inversion recovery magnitude (Figure 3).

Flow cytometric immunophenotyping demonstrated approximately 40% abnormal precursor B lymphoblasts expressing CD19, CD10, CD22, CD34, cCD79a, and HLA-DR, consistent with common B-cell acute lymphoblastic leukemia. Fluorescence *in situ* hybridization was negative for BCR::ABL1, ETV6::RUNX1, KMT2A rearrangement, and iAMP21. Next-generation sequencing identified a pathogenic KRAS p.G13D mutation and a CSF3R c.2426delinsTT (p.Pro809LeufsTer7) frameshift variant, which was reported by the testing laboratory as having potential clinical significance. Cytogenetic analysis demonstrated high hyperdiploidy, while IKZF1 deletion testing was negative.

Pharmacogenetic testing was also performed to assess potential susceptibility to treatment-related toxicities. Genetic testing for steroid-induced osteonecrosis of the femoral head identified the PAI-1 promoter 4G/4G genotype and the ABCB1 C3435T CC genotype, which were classified by the testing report as a high-risk genotype profile for steroid-induced osteonecrosis of the femoral head. Pharmacogenetic testing for commonly used medications in childhood ALL identified heterozygous variants in MTHFR C677T (CT), CEP72 rs924607 (CT), and NUDT15 C415T (CT).

### Definitive diagnosis

Based on the patient's clinical presentation, bone marrow examination, immunophenotypic profile, cytogenetic studies, and molecular analyses, a diagnosis of common B-cell acute lymphoblastic leukemia (B-ALL) was established. Diffuse leukemic bone marrow infiltration was present, together with extensive pathological compression fractures throughout the cervical, thoracic, and lumbar spine. No circulating blasts were detected in the peripheral blood.

### Treatment and follow-up

Following confirmation of the diagnosis, treatment was initiated according to the South China Children's Cancer Group (SCCCG)-ALL-2023 protocol (a multicenter prospective cohort study, registration number ChiCTR2300075242). A prednisone prephase was administered from November 19 to November 25, with aggressive hydration for tumor lysis syndrome prophylaxis, ceftriaxone for empirical antimicrobial coverage, analgesia, calcium supplementation, and vitamin replacement.

The patient's back pain and mobility improved rapidly after treatment. By the fifth day of the prednisone prephase, he could stand independently without neurological deficits. Cerebrospinal fluid (CSF) obtained during the first intrathecal chemotherapy showed no evidence of leukemic involvement. CSF routine and biochemical testing were unremarkable, and flow cytometry did not identify abnormal precursor B lymphoblasts, although the number of evaluable cells was limited.

The VDLD induction regimen (vincristine, daunorubicin, L-asparaginase, and dexamethasone) was initiated on November 26, immediately following the prednisone prephase. No major treatment-related complications occurred during induction. An individualized thoracolumbar brace was provided by the orthopedic team. Bedside rehabilitation included passive joint mobilization, active limb exercises, neuromuscular electrical stimulation, and gradual gait training.

On December 3, 2025 (day 15 of SCCCG-ALL-2023 protocol therapy), bone marrow flow cytometry detected 0.1% residual abnormal B lymphoblasts. According to the SCCCG-ALL-2023 risk-stratification criteria, a day 15 bone marrow MRD level of 0.1%–10% constitutes an intermediate-risk criterion; accordingly, the patient was classified as intermediate risk. The patient completed induction with the VDLD regimen on December 20, 2025. On December 31, 2025, bone marrow morphology showed M1 status (1.5% lymphoblasts), consistent with the conventional definition of morphologic remission (6), while flow cytometric MRD and Ig-NGS were negative. He subsequently received two courses of CAM chemotherapy [cyclophosphamide, cytarabine (Ara-C), and mercaptopurine] from January to February 2026. At reassessment on March 16, 2026, bone marrow flow cytometric MRD and Ig/TCR rearrangement testing remained negative, confirming continued MRD negativity.

Intermediate-risk consolidation included four courses of HD-MTX beginning March 23, 2026. During the fourth course, an elevated methotrexate concentration required leucovorin rescue. Thoracolumbar MRI on June 3 showed persistent multilevel vertebral compression and pathological fractures, with further flattening of some vertebral bodies; hip MRI showed no definite femoral-head osteonecrosis (Supplementary Figures S1 and S2). VALD plus L-asparaginase followed by CAM was administered from June 4 to July 26, followed by blinatumomab from August 19 to September 1. MRI on September 2 showed persistent but overall stable thoracic and lumbar vertebral abnormalities, with slightly more conspicuous pubic symphysis signal and small bilateral hip effusions. Despite persistent radiographic abnormalities, back pain completely resolved and independent ambulation was regained. No leukemia relapse was documented during available follow-up.

The patient's parents reported marked improvement in his back pain and mobility after treatment was initiated. They appreciated the multidisciplinary care provided during hospitalization and expressed confidence in the ongoing treatment and follow-up plan.

## Discussion

### Distinctive clinical presentation and diagnostic challenge

Extensive vertebral compression fractures involving the cervical, thoracic, and lumbar spine caused severe back pain and loss of independent walking. Although bone pain is well recognized in childhood ALL and more commonly involves the long bones or joints (3, 4), extensive multilevel vertebral collapse is rare. Kayser et al. (5) described a 7-year-old boy who presented with severe back pain and multiple vertebral compression fractures despite a normal peripheral blood count. The diagnosis of B-cell ALL was established only after bone marrow examination (5). Reports of children with this pattern of presentation remain scarce.

The distinctive feature of the present case was not bone pain alone, but the combination of an acute trauma-like onset, severe inability to walk, extensive multilevel vertebral compression fractures, and relatively inconspicuous peripheral blood findings without circulating blasts.

The initial presentation posed a diagnostic challenge. The abrupt onset of severe back pain immediately after a minor fall made an acute traumatic spinal injury a reasonable initial consideration. In such a trauma-oriented presentation, the first-line evaluation is generally directed toward identifying structural osseous injury (7). However, persistent severe pain and inability to walk despite conservative treatment prompted further evaluation.

The imaging findings also contributed to the diagnostic challenge. The initial lumbar CT was reported as showing no definite fracture or dislocation, but the original images were unavailable for retrospective review; therefore, a true negative result cannot be established. The available CT obtained at the second hospital allowed retrospective comparison with corresponding MRI. At L4, the vertebral abnormality was relatively inconspicuous on CT but more readily appreciable on MRI as abnormal marrow signal (Figure 1). This comparison illustrates the complementary information provided by CT and MRI and supports MRI when severe or persistent symptoms are not adequately explained by initial imaging (8).

The musculoskeletal presentation was also not truly isolated. At referral to our pediatric center, the patient had leukopenia with neutropenia and prominent cervical and postauricular lymphadenopathy, although no circulating blasts were identified. In retrospect, persistent severe skeletal pain, leukopenia with neutropenia, and lymphadenopathy provided additional clues to a systemic hematologic disorder, though these findings were nonspecific.

### Diagnostic implications

This case highlights several practical considerations for the diagnostic evaluation of children with severe musculoskeletal symptoms. First, the severity and persistence of symptoms should be assessed in relation to the presumed traumatic event. Severe or progressive axial pain, inability to walk, or persistent functional impairment after minor trauma should prompt reassessment when the clinical course is atypical for uncomplicated injury.

Second, clinical-imaging discordance should trigger reconsideration of the initial diagnosis. In the present case, the severity and persistence of symptoms were not adequately explained by the initial trauma-focused assessment. MRI subsequently demonstrated diffuse marrow abnormalities and extensive pathological vertebral compression fractures involving multiple spinal regions. CT is useful for evaluating vertebral morphology and cortical osseous abnormalities, whereas MRI provides more detailed characterization of marrow signal abnormalities (8). Accordingly, MRI should be considered when severe or persistent musculoskeletal symptoms are not adequately explained by radiographs or CT findings (8).

Third, unexplained leukopenia or neutropenia and persistent lymphadenopathy should broaden the differential diagnosis beyond primary orthopedic or rheumatologic disorders. Peripheral blood findings in childhood ALL are heterogeneous, and absence of peripheral blasts does not exclude substantial bone marrow involvement (9). Thus, persistent severe skeletal symptoms accompanied by unexplained cytopenia or lymphadenopathy should prompt consideration of bone marrow examination even without leukocytosis or circulating blasts.

The case also underscores the potential diagnostic consequences of corticosteroid exposure. The patient received methylprednisolone for two days before referral, with transient improvement in back pain. Although the exposure was brief, prediagnostic corticosteroid exposure may reduce leukemic blast burden and complicate diagnostic assessment (10). Therefore, when leukemia is suspected, hematologic evaluation should precede prolonged corticosteroid treatment when clinically feasible.

### Clinical implications and management

When severe musculoskeletal symptoms are not adequately explained by radiographs or CT, whole-spine MRI may help identify diffuse marrow infiltration and occult vertebral fractures. If MRI abnormalities coexist with unexplained cytopenia or persistent clinical suspicion, bone marrow examination should follow without unnecessary delay (3, 9). In this patient, MRI demonstrated extensive vertebral compression fractures without cord compression or neurological deficit, showing that normal neurological findings do not exclude substantial vertebral involvement. Longitudinal skeletal surveillance should assess pain, motor function, vertebral remodeling, spinal alignment, bone mineral density, and treatment-related complications.

The patient carried PAI-1 4G/4G and ABCB1 C3435T CC genotypes that have been investigated in relation to corticosteroid-associated osteonecrosis (11, 12). These findings did not alter leukemia-directed therapy but supported continued skeletal surveillance (13). Additional heterozygous variants in NUDT15 C415T, CEP72 rs924607, and MTHFR C677T were identified. NUDT15 variation is an established determinant of thiopurine-related myelosuppression (14), whereas the clinical relevance of CEP72 and MTHFR variants remains less certain (15). The elevated methotrexate concentration during consolidation cannot be causally attributed to the MTHFR genotype. These findings should therefore be regarded as background information rather than evidence supporting individualized treatment modification.

### Additional molecular findings

The CSF3R c.2426delinsTT (p.Pro809LeufsTer7) frameshift variant identified in this patient was an unusual finding given the presence of neutropenia at diagnosis. CSF3R encodes the granulocyte colony-stimulating factor receptor (G-CSFR), a key regulator of granulopoiesis, and truncating or activating CSF3R alterations are classically associated with severe congenital neutropenia (SCN) and myeloid neoplasms (16). In SCN, acquired truncating CSF3R mutations have been associated with clonal evolution and progression to myeloid malignancies (16, 17). CSF3R alterations have also been rarely reported in B-ALL, although their biological and clinical significance in B-lineage leukemia remain incompletely understood (18, 19). The occurrence of a CSF3R alteration in B-ALL therefore represents an unusual molecular finding, but its presence alone does not establish a pathogenic role in leukemogenesis.

In this patient, no functional validation, paired germline analysis, or longitudinal assessment of variant allele burden was performed. Therefore, the available evidence is insufficient to determine whether the variant is somatic, germline, or directly involved in leukemogenesis. Although its coexistence with neutropenia is noteworthy, there was no documented history of congenital or persistent neutropenia before leukemia diagnosis, and extensive leukemic marrow involvement provides a more immediate explanation for impaired normal hematopoiesis. The CSF3R finding did not alter the diagnosis or treatment of B-ALL. The leukemic population showed a precursor B-cell immunophenotype without a myeloid lineage-defining phenotype, and current classification requires appropriate immunophenotypic evidence for lineage assignment (20–22). Thus, the CSF3R variant should currently be regarded as an incompletely characterized molecular finding rather than evidence of mixed-lineage disease or an indication for targeted therapy.

The high-hyperdiploid cytogenetic background and the accompanying KRAS p.G13D mutation also provide relevant genomic context for this case. High hyperdiploidy is a common genetic subtype of childhood B-ALL and is generally associated with favorable clinical features and outcome. Genomic studies have shown that alterations of the RTK/RAS pathway, including KRAS, are frequent secondary events in high-hyperdiploid ALL (23, 24). In this patient, however, the specific contribution of the KRAS p.G13D mutation or the high-hyperdiploid state to the extensive vertebral compression fractures cannot be established. We therefore regard these findings as part of the biological context of the leukemia rather than as evidence for a specific mechanism underlying the skeletal manifestations.

### Limitations

This report has several limitations that should be considered when interpreting its findings. First, the original CT images from the first hospital were unavailable for retrospective review. Therefore, we cannot determine whether subtle vertebral or marrow abnormalities were already present on that examination or whether the findings were simply not considered diagnostically significant at the time. The CT-MRI comparison presented in Figure 1 is consequently intended to illustrate the complementary diagnostic information provided by the two modalities rather than to establish that the initial CT was falsely negative.

Second, serum parathyroid hormone (PTH) was not measured at presentation, limiting a more complete assessment of calcium-phosphate homeostasis and potential metabolic contributors to skeletal fragility. Dedicated acid-base assessment was also not performed. In addition, bone turnover biomarkers and direct assessments of the bone marrow microenvironment were unavailable, limiting mechanistic evaluation of the severe skeletal phenotype.

Moreover, the clinical significance of the CSF3R p.Pro809LeufsTer7 variant remains uncertain because functional, germline, and longitudinal variant analyses were unavailable. Similarly, the pharmacogenetic findings identified in this patient cannot be used to infer individual treatment responses or toxicity risk beyond their currently established clinical evidence.

Finally, although the patient achieved remission and regained independent ambulation, the duration of follow-up remains limited. Continued surveillance is required to assess long-term vertebral remodeling, bone mineral density, skeletal growth, spinal deformity, functional recovery, and treatment-related skeletal complications. Larger multicenter cohorts and longitudinal studies will be needed to determine the mechanisms, prognostic implications, and long-term skeletal outcomes of extensive vertebral involvement in childhood ALL.

## Conclusion

This case highlights that childhood B-cell acute lymphoblastic leukemia may first present with extensive vertebral compression fractures, despite limited peripheral blood abnormalities and the absence of circulating blasts. In children with persistent axial skeletal pain or unexplained vertebral compression fractures, hematologic malignancy should remain an important differential diagnosis, and early MRI followed by timely bone marrow examination should be considered when clinical suspicion persists. Recognizing this uncommon presentation may facilitate earlier initiation of treatment.

## Data Availability

The raw data supporting the conclusions of this article will be made available by the authors, without undue reservation.
