# Supplementary material for: Extensive vertebral compression fractures as the initial manifestation of childhood B-cell acute lymphoblastic leukemia despite limited peripheral blood abnormalities: a case report
Source: Front Pediatr. 2026 Oct 12;14:1945539. doi: 10.3389/fped.2026.1945539 (PMC13696464; doi:10.3389/fped.2026.1945539)
Supplement: Supplementary file 1 [file Supplementaryfile1.docx]

**Supplementary Table 1.** Timeline of the clinical presentation, diagnostic workup, treatment, and follow-up.

| **Date** | **Clinical course and key findings** |
| --- | --- |
| **Nov 11, 2025** | Fall followed by severe back pain. |
| **Nov 12, 2025** | Initial evaluation at a local hospital. Lumbar CT reportedly showed no definite vertebral fracture or dislocation, and a soft-tissue injury was initially diagnosed. |
| **Nov 14–16, 2025** | Evaluation at a second hospital. Abdominal CT included the lumbar spine, while lumbar MRI demonstrated flattening of the T11–L5 vertebral bodies and abnormal signal intensity in T12. |
| **Nov 17–18, 2025** | Admission to our pediatric center for persistent severe back pain and inability to walk. Laboratory tests showed leukopenia, neutropenia, and elevated hs-CRP, without circulating blasts. Bone marrow aspiration demonstrated marked lymphoblast infiltration, with lymphoblasts accounting for 71% of nucleated cells in the sternal marrow and 59% in the iliac marrow. |
| **Nov 19–25, 2025** | Prednisone prephase was initiated according to the SCCCG-ALL-2023 protocol. Whole-spine MRI demonstrated diffuse bone marrow infiltration and extensive pathological compression fractures involving the cervical, thoracic, and lumbar spine. Back pain and mobility improved rapidly during the early treatment period, and the patient was able to stand with assistance. |
| **Nov 26–Dec 20, 2025** | VDLD induction chemotherapy was initiated according to the SCCCG-ALL-2023 protocol. Day-15 bone marrow assessment showed 0.1% residual abnormal B lymphoblasts by flow cytometry; the patient was classified as intermediate risk according to the protocol. |
| **Dec 31, 2025** | Bone marrow morphology showed M1 status (1.5% lymphoblasts), while flow cytometric MRD and Ig-NGS were negative. |
| **Jan 4–Feb 19, 2026** | Two courses of CAM chemotherapy were administered as part of intermediate-risk therapy. |
| **Mar 16, 2026** | Bone marrow reassessment showed negative flow cytometric MRD and Ig/TCR rearrangement testing. |
| **Mar 23–May 17, 2026** | Intermediate-risk consolidation chemotherapy, including four courses of high-dose methotrexate (HD-MTX), was administered. During the fourth HD-MTX course, an elevated methotrexate concentration required leucovorin rescue. |
| **Jun 3, 2026** | Follow-up thoracolumbar MRI demonstrated persistent multiple vertebral compression/pathological fractures, with further flattening of some vertebral bodies compared with the initial examination. Hip MRI showed no definite evidence of femoral-head osteonecrosis. |
| **Jun 4–Jul 26, 2026** | VALD plus L-asparaginase followed by CAM chemotherapy was administered. Back pain completely resolved, and independent ambulation was regained. |
| **Aug 19–Sep 1, 2026** | Blinatumomab therapy was administered. No leukemia relapse was documented during the available follow-up period. |
| **Sep 2, 2026** | Follow-up thoracolumbar MRI showed persistent multiple thoracic and lumbar vertebral compression fractures, overall similar to the June 3 examination. Abnormal signal at the pubic symphysis was slightly more conspicuous, with small bilateral hip joint effusions. |

Abbreviations: ALL, acute lymphoblastic leukemia; CT, computed tomography; MRI, magnetic resonance imaging; MRD, measurable residual disease; Ig-NGS, immunoglobulin next-generation sequencing; TCR, T-cell receptor; VDLD, vincristine, daunorubicin, L-asparaginase, and dexamethasone; CAM, cyclophosphamide, cytarabine, and 6-mercaptopurine; HD-MTX, high-dose methotrexate.

**Supplementary Figure S1.** Serial MRI follow-up of persistent thoracolumbar vertebral abnormalities.

A1-C1: T1-weighted (A1), T2-TIRM (B1), and T2-weighted (C1) images obtained on June 3, 2026. A2-C2: Corresponding T1-weighted (A2), T2-TIRM (B2), and T2-weighted (C2) images obtained on September 2, 2026. Multiple thoracic and lumbar vertebral compression/pathological fractures remained visible on follow-up MRI, with overall similar findings between the two examinations.

**Supplementary Figure S2.** Follow-up magnetic resonance imaging of the bilateral hip joints.

Representative coronal T2-weighted fat-suppressed magnetic resonance images obtained on (A) June 3, 2026 and (B) September 2, 2026. The bilateral hip joints remained aligned on both examinations, with no definite femoral-head osteonecrosis reported. Small bilateral hip joint effusions were noted.
